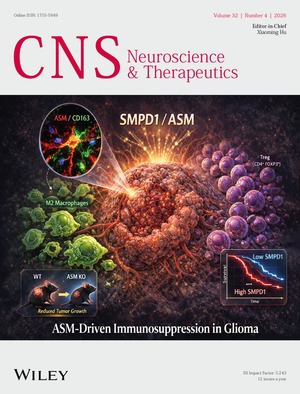# Front Cover

**DOI:** 10.1002/cns.70863

**Published:** 2026-04-08

## Abstract

The cover image is based on the article *SMPD1 as a Potential Prognostic Biomarker in Glioma Is Associated With an Immunosuppressive Microenvironment* by Yanan Xu et al., https://doi.org/10.1002/cns.70813.